# Analecta Minora; or Minor Periscope

**Published:** 1826-04

**Authors:** 


quences would be again suspended. Similar experiments were made with white arsenic, the upas tiente, and Prussic acid. Eight grains of arsenic were introduced into a wound made in the thigh of a dog. Three quarters of an hour afterwards the glass was applied, and kept exhausted for four hours. The dog experienced no inconvenience. Another dog was similarly treated, but no glass applied, and the animal died in fifteen hours. Six drops of Prussic acid were infused into a small wound in the thigh of a rabbit. The glass was immediately applied, and kept so for twelve minutes. The animal felt no bad effects.—The glass was removed, and quickly the rabbit was seized with convulsions and was supposed to be dying. The re-application of the glass was soon followed by a restoration of the pristine state of the animal. After twelve minutes the glass was again removed, and the convulsions and other bad symptoms soon reappeared and required the third application of the exhauster. The rabbit could not dispense with the glass till after half an hour from the introduction of the poison. The same process was instituted on another rabbit, but without the exhauster. The animal died in two minutes. Experiments of a similar kind were made with the upas tiente, and with analogous results. It appears evident that the cupping glass prevents the poison from finding its way into the system, and that thus the process may be of considerable importance in the healing art.—*Rev. Medicale.*

P. S. More recently Dr. Barry has read a memoir on this subject before the Medico-Chirurgical Society of London, which excited long discussions during two sittings of the Society. The evidences of the efficacy of cupping-glasses in preventing the absorption of poisons were irresistible, and the opinion of the Society was unanimous respecting the merits of Dr. Barry's experiments. It has been proved lately in France, that the bites of vipers, both on man and inferior animals, were rendered entirely harmless, by the application of cupping-glasses.

---

2. *Croup.* Mr. Pretty, an intelligent and very observant practitioner of this metropolis, has thrown out some valuable hints on this disease, in a paper read before the Medico-Chirurgical Society, and published in the January number of the Medical and Physical Journal. He has never had any reason to think that genuine idiopathic croup possessed contagious properties. But he has witnessed many severe and fatal cases of croup, after, or rather in conjunction with, simple scarlet fever and malignant sore throat, in which it was contagious—" but not as the primary disease." It seemed be owing to an extension of inflammation from the fauces to the larynx and trachea in young children. It generally proved fatal. On dissection, he found the usual products of inflammation—the adventitious membrane—the copious exudation of thick mucus—and, in one case, ulceration in the interior of the larynx. Mr. Pretty makes some pertinent observations on what is described by the late Dr. Clarke, as a peculiar convulsive disease of children, and which has been termed " Cerebral Croup." Mr. Pretty's attention was particularly drawn to this disease by its occurrence in two or three of his own children, one of whom, having been, for several weeks, affected, at indefinite periods, with paroxysms of spasms, accompanied by croupy inspiration, was seized with two slight convulsions in the morning, and a third in the afternoon, which terminated the child's existence. On dissection, the blood-vessels of the brain were found loaded, with watery effusion into the ventricles, and between the membranes. Except some intus-susceptions in the intestines, there was no other mark of disease. The second child (they were twins) was seized with a convulsion the week after her sister's death, which was followed by repeated paroxysms of croupy and impeded respiration, threatening a repetition of the convulsion. Symptoms of meningitis, and even of effusion supervened, as startings, screamings, insensibility, squinting, dilated pupils, &c. which formidable symptoms were removed by leeches, blisters, and calomel purgatives. The croupy paroxysms continued for several months, and ultimately yielded to assafœtida. A third child was seized in a similar manner, and the complaint was removed by the same means as above described; but the croupy spasms continued for some time, till country air and a good nurse restored him to health. Mr Pretty has seen upwards of a dozen of similar instances, and in all of them he had reason to believe the head was the primary seat of the complaint.

Undoubtedly it is to the brain and nervous system we are to look for the immediate cause of convulsion of muscles; but then it is not always an *inflammatory condition* of the brain which obtains on these occasions. Irritation in the stomach or bowels, will cause a convulsion much more frequently and readily than meningitis. We have seen a considerable number of those spasmodic affections of the muscles about the glottis, producing a croupy kind of inspiration, very alarming to those who are not acquainted with its nature. In almost all these cases we have found fever or symptoms of inflammation *absent*—the whole depending either on the irritation of teething, worms, or disordered secretions in the bowels. Purgatives, followed by any gentle antispasmodic, relieve the complaint. Is it not evident that this croupy inspiration, in Mr. Pretty's second child, was independent of any congestive or inflammatory condition of the brain, when it gave way to assafœtida after resisting all other means?—It is proper, of course, to pay attention to the head in all convulsive affections; but we repeat our conviction that the croupy inspiration of children is, in itself, no proof, or even indication of fulness of blood in the brain. When there are general convulsions or any *other* symptoms of cerebral affection, then we would not be slow in unloading the vessels of the brain.—For many important observations on croup and convulsions, we refer to Mr. Pretty's paper, in the Journal abovementioned.

---

3. *Milky Blood.* Dr. Venables has addressed something like a reclamation to our worthy friends of the Repository, in consequence of a passage in the "Clinical Remarks" of the February Number of the above Journal, where the reporter notices a milky or whey-coloured appearance of the blood drawn from a patient—a phenomenon that he had not seen recorded before. Dr. V. claims priority on this point, having noticed the fact in his work on dropsy—and drawn a conclusion that the said phenomenon was an indication of inflammation in the blood. In respect to the originality of this discovery, we may observe that Hewson has dedicated a whole chapter to the subject in question, quoting instances out of number, from Morgagni down to his own time. This celebrated anatomist and physiologist, however, found the milky appearance of the blood obtain under so many opposite states of the subject from whom the blood was drawn, that he was put to his wit's ends to account for the phenomenon. Inflammation he places quite out of the question, and attempts not the explanation on that ground. He admits that blood drawn from people soon after eating, sometimes exhibits the milky or whey-like appearance; but, he is more inclined, upon the whole, to attribute the phenomenon to the presence of oily particles in the blood, from a too rapid absorption of fat.

Mr. Cullen of Sheerness, published a paper on this subject, some fifteen years ago, and sent a sample of the milky blood to Mr. Brookes, the anatomist, at whose house it was seen for many months afterwards, still preserving its milky and fluid state. Enough has been said to satisfy Dr. Venables that he was not wronged by the Clinical Reporter, in our cotemporary—and that the reporter himself was rather defective in memory, at the moment he wrote the observation above alluded to. We have seen three instances of this kind—and in all three, the patients had been eating food three or four hours before the blood was drawn. As bleeding is seldom employed, except where there is some local inflammation or general excitement, the milky appearance in question will, of course, be commonly observed cotemporaneous with inflammation. But this, by no means proves that the former is an indication of the latter. When we consider how common is inflammation, and how rarely do we see the milky appearance of the blood, we are not at all authorised to connect them together by any link of necessary dependency.

---

4. *Otitis Acuta.* Dr. James Kennedy, the able physician and pathologist, has drawn the attention of his brethren to a modification of treatment in this very painful disease. He was led to this mode of treatment so far back as 1816, while attending a vigorous young woman labouring under acute ear-ache. After sanguineous depletion, an emetico-cathartic medicine was exhibited, which acted powerfully, sursum ac deorsum. The ear-ache permanently disappeared during the operation of the medicine. Since the above period, Dr. Kennedy has treated twenty-five cases of the same disease, in the same manner, and with the same success. More recently he was led, for the sake of simplicity in practice, to try the plan in question, without blood-letting—and sixteen cases were treated by the emetico-cathartic remedy alone, with perfect success. In eleven instances, the emetic was twice, and in five, thrice exhibited. "In every one of them the convalescence was rapid and perfect." The general formula was, 30 grains of ipecacuan, one grain of tartrite of antimony, and five grains of calomel—taken in a little treacle, and followed by a free draught of tepid water. We refer to the last Number (March) of our cotemporary (the Repository) for many interesting remarks and details in Dr. Kennedy's paper, for which we have not space here. Our able author believes that this mode of treatment will be found very beneficial in erysipelatous inflammation (which is so often connected with derangement of the digestive organs) gout, and some other affections of a kindred nature. The paper deserves serious attention.

---

5. *Epileptic Convulsions.* Dr. Blake, surgeon of the 7th Dragoon Guards, has related (*Med. and Phys. Journ. January,* 1826,) the case of a young soldier who received a blow from the clenched fist of a comrade on the centre of the *right* parietal bone, February 4th, 1824. On the 8th, he was received into hospital with the common symptoms of fever, which gave way to the usual antiphlogistic treatment in a few days. But head-ache remained, and increased in violence, with foul tongue and slow pulse. Purgation, blisters, bleeding, local and general, together with mercury, so as to affect the system, removed the head-ache, and the man was discharged to duty on the 24th. On the 29th, he returned, complaining of pain over the crown of the head, with slow pulse and dilated pupils. The same treatment as before, but not with the same success. On the 2d March, he was seized, at 2 o'clock, with a fit of *epileptic convulsions*, on recovering from which, he was found to have hemiplegia of the *left* side. The epileptic fits were soon after renewed, and they continued, with little intermission, till 7 o'clock, when the symptoms were so urgent as to induce Dr. Blake, in consultation, to trephine the site of the original injury. The bone was very thick, and but little adhesion existed between it and the subjacent dura mater. There was no effusion, nor any appearance of disease. As soon as the piece of bone was removed, the convulsions became mitigated, and, in a few hours, they ceased altogether. In about a month the paralysis disappeared, and the man was discharged.

We do not, in the slightest degree, object to the operation which Dr. Blake had recourse to, under the threatening symptoms which existed; but we greatly doubt whether the conclusion is legitimate that the cessation of the epileptic paroxysm, a few hours after the application of the trephine, was the effect of that operation. We all know that a paroxysm of this kind will continue for many hours, and cease spontaneously, without a portion of cranium being removed And, if we demur to the legitimacy of Dr. Blake's *post hoc, ergo propter hoc* conclusion, we object still more strongly to the explanation which he attempts of the modus *operandi*, or rather *sanandi*, of the trephine on this occasion. That there was pressure on some portion of the brain, we admit, because the hemiplegia was the best *living* proof which could be adduced; but that the admission of atmospheric pressure from *without* (if, indeed, there was any atmospheric pressure admitted) should relieve the sanguineous pressure from *within*, is to us as incomprehensible as the reasoning of the rustic, who argued that Tenterden steeple was the cause of the Goodwin Sands.

P. S. We have been obliged to postpone, till next Number, a large mass of matter collected for the Periscope and the Analecta Minora, in consequence of papers having over-run their calculated dimensions. As it is, we give nearly 60 pages over the prescribed limits of the Number.

---

